# The mitogenome of the southern Brook Lamprey, *Ichthyomyzon gagei* (Cyclostomata: Petromyzontidae)

**DOI:** 10.1080/23802359.2016.1258347

**Published:** 2017-01-04

**Authors:** Rex Meade Strange, Vivian T. Truong, Kimberly J. Delaney

**Affiliations:** Department of Biology, University of Southern Indiana, Evansville, IN, USA

**Keywords:** Lamprey, *Ichthyomyzon gagei*, mitochondrial DNA

## Abstract

The southern brook lamprey (*Ichthyomyzon gagei*) is a non-parasitic lamprey endemic to the southeastern US. Here, we report the complete mitogenome of this basal vertebrate and found its genomic organization to be similar to that of other reported lamprey mitogenomes. Nucleotide sequence identities for individual proteins range from 90% to 94% when compared with the congeneric species *I. fossor* and *I. unicuspis*. Finally, phylogenetic analysis revealed *I. gagei* to be sister to these other species of *Ichthyomyzon.* These genomic data provide a baseline for future investigations regarding the molecular evolution of basal vertebrates.

The lampreys are important model organisms in evolutionary studies because of their peculiar life histories and their retention of many features reminiscent of the earliest vertebrates (Osorio and & Retaux 2008). Although many lamprey species actively feed on other fishes as juveniles before maturation, the larva of non-parasitic species transform directly into non-feeding adults, spawn, and die. This parasitic/non-parasitic dichotomy is reflected in the taxonomic diversity of lampreys, in which most parasitic species are paired with a non-parasitic derivative (Pereira et al. 2010). Although such ‘satellite’ species occur in most genera of lampreys, the three species pairs of the genus *Ichthyomyzon* exquisitely illustrate this pattern.

The southern brook lamprey (*Ichthyomyzon gagei*) is a non-parasitic species that is considered to be a derivative of the parasitic chestnut lamprey (*I. castaneus*), with which it co-occurs in the southeast United States (Warren & Burr 2014). The life history of *I. gagei* has been summarized, its karyotype has been described (Howell & Duckett 1971), and its phylogenetic relationships have been inferred from mitochondrial cytochrome *b* sequences (Lang et al. 2009).

We report herein the mitochondrial genome of *Ichthyomyzon gagei* and its phylogenetic relationship as inferred from the entire mitogenome sequence. The specimen was an adult male collected from Cadron Creek, Arkansas, (35.1936N; 92.5302W), ethanol fixed, and vouchered at the University of Southern Indiana Natural History Collection (# R2146). DNA was extracted from muscle tissue following conventional proteinase K and phenol–chloroform extraction protocols. Mitochondrial DNA was amplified by primer-walking and the individual PCR fragments were sequenced.

The mitogenome of *Ichthyomyzon gagei* (Genbank: KY056640) is 16,359 bp in length and is slightly longer than those reported for *I. fossor* (16,150 bp) and *I. unicuspis* (16,163 bp). Most of the length differences are due to variation in the non-coding regions (NC1 and NC2), although occasional insertions/deletions were found in tRNA and rRNA genes. Base composition (L-strand) was similar to that previously reported for other vertebrates with a low guanine content (12.8%) and almost equal frequencies of adenine (33.1%), cytosine (23.8%) and thymine (30.3%) residues. The mitogenome has the same genic arrangement as *Petromyzon marinus* (Lee & Kocher 1995) and includes 13 protein-coding genes, 2 ribosomal RNAs, 22 transfer RNAs and 2 non-coding regions. The percent identities of the protein-coding genes from *I. gagei* and the two other available species of *Ichthyomyzon* ranged from 90% (ND4) to 94% (CO1, CO2, ATP8, CO3, and ND3). Comparison with *P. marinus* revealed greater divergences (81% ND3 to 89% CO2 and CO3).

Phylogenetic analyses of the 16 available lamprey mitogenomes were consistent with previous relationships inferred from mitogenomic data (Lang et al. 2009; Ren et al. 2015; Figure 1). *Ichthyomyzon gagei* is sister to *I. fossor* and *I. unicuspis*, while the three available species of the genus *Ichthyomyzon* form the sister group to the monotypic genus *Petromyzon*. The remainder of petromyzontid lampreys form a well-supported group that is sister to the *Petromyzon* +* Ichthyomyzon* clade.

**Figure 1.**
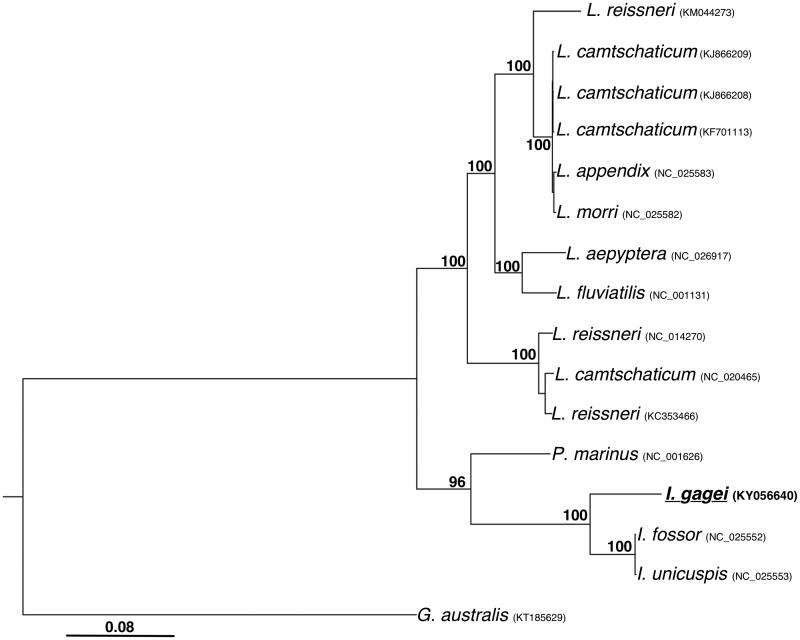
Phylogenetic analysis of lamprey mitogenomes. Full length mitogenome sequences were aligned using MUSCLE (Edgar 2004). Tree was inferred from both maximum parsimony (PAUP*; Swofford 2002) and maximum likelihood analysis with a HKY85 substitution model (PhyML; Guindon et al. 2010). Bootstrap values (1000 replicates) are indicated at each node. The current species under investigation (*I. gagei,* Genbank: KY056640) is underlined and bold. GenBank accession numbers are included for each sequence.
